# Analysis of the Interferon Gamma (rs2430561, +874T/A) Functional Gene Variant in Relation to the Presence of Cardiovascular Events in Rheumatoid Arthritis

**DOI:** 10.1371/journal.pone.0047166

**Published:** 2012-10-15

**Authors:** Mercedes García-Bermúdez, Raquel López-Mejías, Carlos González-Juanatey, Alfonso Corrales, Gema Robledo, Santos Castañeda, José A. Miranda-Filloy, Ricardo Blanco, Benjamín Fernández-Gutiérrez, Alejandro Balsa, Isidoro González-Alvaro, Carmen Gómez-Vaquero, Javier Llorca, Javier Martín, Miguel A. González-Gay

**Affiliations:** 1 Instituto de Parasitología y Biomedicina López-Neyra, Consejo Superior de Investigaciones Científicas, IPBLN-C.S.I.C., Granada, Spain; 2 Department of Rheumatology, Hospital Universitario Marqués de Valdecilla, IFIMAV, Santander, Spain; 3 Cardiology Division, Hospital Xeral-Calde, Lugo, Spain; 4 Department of Rheumatology, Hospital Universitario La Princesa, IIS-Princesa, Madrid, Spain; 5 Department of Rheumatology, Hospital Xeral-Calde, Lugo, Spain; 6 Department of Rheumatology, Hospital Clínico San Carlos, Madrid, Spain; 7 Department of Rheumatology, Hospital Universitario La Paz, Madrid, Spain; 8 Department of Rheumatology, Hospital Universitario de Bellvitge, IDIBELL, ĹHospitalet del Llobregat, Barcelona, Spain; 9 Department of Epidemiology and Computational Biology, School of Medicine, University of Cantabria, and CIBER Epidemiología y Salud Pública (CIBERESP), IFIMAV, Santander, Spain; Tor Vergata University of Rome, Italy

## Abstract

**Objective:**

Rheumatoid arthritis (RA) is a chronic inflammatory disease associated with increased cardiovascular (CV) morbidity and mortality. Since interferon-gamma (IFN-γ) has a direct effect on inflammation, in this study we assessed the potential association of the *IFNG* functional gene variant rs2430561 with CV disease in patients with RA.

**Methods:**

One thousand six hundred and thirty-five patients fulfilling the 1987 American College of Rheumatology classification criteria for RA were genotyped for the *IFNG* (rs2430561, +874T/A) gene polymorphism using TaqMan genotyping assay. Patients were stratified according to the presence of CV events or not. Logistic regression models to explain the presence of CV disease according to the *IFNG* rs2430561 allele distribution were performed. The potential influence of this variant in the development of subclinical atherosclerosis was also analyzed in a subgroup of patients with no history of CV events to determine carotid artery intima-media thickness (IMT) (n = 286) and presence of carotid plaques. Levels of the cytokine were determined in a subgroup of patients by ELISA.

**Results:**

Adjusted logistic regression model disclosed that presence of the minor allele A was not associated with increased risk of suffering CV events in RA patients. Besides, differences did not achieve statistical significance regarding carotid IMT and presence of carotid plaques in RA patients carrying *IFNG* rs2430561 variant allele. Levels of IFN-γ were higher in patients who had suffered CV events compared to patients who did not.

**Conclusion:**

Our results do not support a role of *IFNG* rs2430561 (+874T/A) functional gene variant in the development of CV disease in RA patients.

## Introduction

Rheumatoid arthritis (RA) is a chronic autoimmune disease associated with high risk of cardiovascular (CV) disease. Atherosclerosis is the leading cause of increased CV morbidity and mortality in RA patients [1]. Besides classic CV risk factors, “high-grade” systemic inflammation and immune dysregulation play a crucial role in the development of the accelerated atherosclerosis observed in RA [1], [2], [3]. With respect to this, atherosclerosis and RA share inflammatory mechanisms that may explain the augmented incidence of CV events observed in patients with RA [3], [4]. Moreover, recent studies have disclosed the importance of genetic factors in the development of CV disease in patients with RA [5], [6], [7], [8], [9], [10].

**Table 1:** Demographic characteristics of the RA patients included in the study.

Variables	N = 1635
Females	1215 (74.31)
Age of patients at the time of disease diagnosis, years, median (IQR)	54 (43–64)
Time follow up, years, median (IQR)	10.2 (5–17)
Anti-CCP positive (n = 1330)	766 (57.59)
Rheumatoid Factor positive (n = 1604)	1106 (68.95)
Cardiovascular events	332 (20.31)
Ischemic heart disease	157 (9.63)
Cerebrovascular accidents	78 (4.78)
Heart failure	91 (5.76)
Peripheral arteriopathy	39 (2.46)
Hypertension (n = 1621)	666 (41.09)
Diabetes mellitus (n = 1616)	216 (13.37)
Dyslipidemia (n = 1619)	643 (39.72)
Obesity (n = 1534)	359 (23.40)
Smoking habit (n = 1552)	447 (28.80)

Except where indicated otherwise, values are n (%). IQR: Interquartile Range. Anti-CCP: Anti-Cyclic Citrullinated Peptide antibodies.

Two T cell-derived cytokines of potential importance in RA are interferon gamma (IFN-γ) and interleukin (IL)-17. Although only small amounts of IFN-γ are produced by synovial T cells, this cytokine serves as a marker for the subset of activated T cells known as Th1 helper cells. These cells promote and amplify autoimmune diseases. IFN-γ also has a direct effect on inflammation by increasing major histocompatibility complex (MHC) class II expression as well as priming macrophages to produce inflammatory and tissue-damaging mediators such as TNF-α, proteases, reactive oxygen species, and nitric oxide [11].

IFN-γ is a pleiotropic soluble cytokine with antiviral and anti-tumor properties. This cytokine is found in RA patientś synovium and synovial fluid [12], and polymorphisms within its gene sequence may be biologically plausible candidates for influencing the incidence and severity of RA [13]. With respect to this, the functional Single Nucleotide Polymorphism (SNP) at position +874 of the *IFNG* gene (rs2430561) [NCBI Reference Sequence: NT_029419.12] maps to a putative nuclear factor-κB (NF-κB) binding site. The presence of T allele in this functional variant improves the NF-κB binding efficiency leading to increased IFN-γ expression *in vitro* [14].

**Table 2:** Differences in genotype and allele frequencies of *IFNG* rs2430561 functional gene variant between RA patients with CV events *versus* patients without CV events.

*IFNG* +874T/A	With CVevents	Without CVevents	*p*	OR [95% CI]
TT	75 (23.08)	307 (24.07)		Ref.
TA	179 (55.08)	653 (52.53)	0.45	1.12 [0.82–1.53]
AA	71 (21.85)	283 (22.77)	0.89	1.03 [0.70–1.50]
TA+AA	250 (76.93)	936 (75.30)	0.54	1.09 [0.81–1.47]
T	329 (50.62)	1267 (50.97)		
A	321 (49.38)	1219 (49.03)	0.87	1.01 [0.85–1.21]

CV: Cardiovascular. Except for OR, values are number (%). OR [95% CI]: Odds Ratio with 95% Confidence Interval.

Association of a polymorphic microsatellite located in the first intron of the *IFNG* gene with susceptibility to RA was reported [15], although other studies failed to confirm the association between the *IFNG* gene polymorphism and RA susceptibility or severity [13], [16]. *IFNG* repeat polymorphism is in complete linkage disequilibrium with the rs2430561 functional variant [14]. Association of *IFNG* gene polymorphism with systemic lupus erythematosus has also been described in Korean population [17] and polymorphisms in the *IFNG/IL-6* gene region may contribute to sex bias in susceptibility to RA [18]. No association was reported at Genome-wide association analysis level for *IFNG* and RA, although a significant association was reported for a variant located near *IFNG* gene and ulcerative colitis [19].

Atherosclerosis is a chronic inflammatory disease. In the general population, in individuals not affected by RA, IFN-γ is highly expressed in atherosclerotic lesions and has emerged as a significant factor in the development and progression of CV disease. IFN-γ is of main importance in the development of oxidative stress for antimicrobial and anti-tumoral defense within the cell-mediated immune response. In CV disease, biochemical reactions induced by interferon-gamma may have detrimental consequences for host cells. IFN-γ is the most important trigger for the formation and release of reactive oxygen species. Chronic reactive oxygen species -production leads to the depletion of antioxidants like vitamin C and E and glutathione, with a consequence that oxidative stress develops. Oxidative stress plays a major role in the atherogenesis and progression of CV disease, and it may also account for the irreversible oxidation of other oxidation-sensitive substances like B-vitamins [20]. Previous studies have also shown that IFN-γ is the main trigger for production and release of reactive oxygen species in endothelium, and a subtype of T cells involved in rheumatoid synovitis and atherosclerosis produce large amounts of IFN-γ [20]. Furthermore, IFN-γ has an important role in atherosclerosis and plaque disruption enhancing expression of adhesion molecules on endothelial cells [21]. IFN-γ can also recruit macrophages and T cells into plaque, contributing to production of reactive oxygen species, inhibiting collagen production, stimulating matrix metalloproteinases, and inducing tissue factor expression [1], [22], [23].

**Table 3:** Logistic regression model to explain the presence of CV disease in patients with RA according to *IFNG* rs2430561 allele distribution.

*IFNG* +874T/A	*p*	OR [95% CI]	*p* *	OR [95% CI]*
A vs. T	0.87	1.01 [0.85–1.21]	0.56	1.08 [0.84–1.39]

* Analyses adjusted for gender, age at rheumatoid arthritis diagnosis, follow-up time from the disease diagnosis, anti-CCP status, and long-standing CV risk factors: hypertension, diabetes mellitus, dyslipidemia, obesity and smoking habit. OR [95% CI]: Odds Ratio with 95% Confidence Interval.

Taking account all of these evidences, the main goal of this study was to assess for first time the potential implication of *IFNG* functional gene variant rs2430561 (+874T/A) in the risk of CV disease of patients with RA.

## Patients and Methods

### Patients and Study Protocol

Between March 1996 and September 2008, 1635 consecutive patients that fulfilled the 1987 American College of Rheumatology classification criteria for RA [24] were recruited from the Rheumatology Outpatient Clinics of Hospital Xeral-Calde (Lugo), Hospital Clínico San Carlos (Madrid), Hospital Universitario La Paz (Madrid), Hospital Universitario de La Princesa (Madrid), Hospital Universitario Bellvitge (Barcelona), and Hospital Universitario Marqués de Valdecilla (Santander), Spain. Patients were assessed for differences in the *IFNG* rs2430561 functional gene polymorphism.

**Table 4:** Comparison of carotid artery intima media thickness (IMT) according to *IFNG* rs2430561 variant.

rs2430561	IMT mm, mean (SD)	*p*
TT (n = 73)	0.74 (0.16)	
TA (n = 153)	0.75 (0.17)	
AA (n = 60)	0.71 (0.17)	
Model		0.33
T (n = 299)	0.74 (0.17)	
A (n = 273)	0.73 (0.17)	0.45

#### Ethics statement

A subject’s written consent was obtained according to the declaration of Helsinki, and the design of the work was approved by the Ethics Committee of the different centers that collaborated in the study.

Between December 2009 and January 2010 patient’s clinical records were examined until patient’s death, loss of follow-up or December 1^st^, 2009. Information on the main demographic and clinical data regarding clinical characteristics of the patients enrolled in the study, classic CV risk factors and CV events of patients for whom clinical information was available at the time of the study (n = 1635) were registered and are shown in **Table 1**.

**Table 5:** Logistic regression model to explain the presence of carotid plaques in patients with RA according to *IFNG* rs2430561 allele distribution.

	*p*	OR [95% CI]	*p* *	OR [95% CI]*
rs2430561 A vs. T	0.60	1.09 (0.78–1.53)	0.25	1.31 (0.83–2.07)

* Analyses adjusted for gender, age at rheumatoid arthritis diagnosis, follow-up time from the disease diagnosis, anti-CCP status and classic cardiovascular risk factors: hypertension, diabetes mellitus, dyslipidemia, obesity and smoking habit. OR [95% CI]: Odds Ratio with 95% Confidence Interval.

Clinical definitions for classic CV risk factors and CV events (ischemic heart disease, heart failure, cerebrovascular accident or peripheral arteriopathy) were established as previously described [5], [25]. A CV event was considered to be present if the patient had ischemic heart disease, heart failure, cerebrovascular accident or peripheral arteriopathy. The definition of ischemic heart disease included acute coronary syndromes with or without persistent ST-segment elevation and chronic coronary heart disease. Ischemic heart disease was diagnosed if any of the following criteria were satisfied: a recorded diagnosis of ischemic cardiopathy, on account of some acute coronary syndrome (acute myocardial infarction or unstable angina), the presence of pathological Q waves in the electrocardiogram, and coronary images showing >50% stenosis of at least one coronary vessel. Data regarding the clinical presentation of heart failure were also collected from all patients. A patient was considered to have a cerebrovascular accident when he/she had a stroke and/or transient ischemic attacks. Strokes were classified according to their clinical features and they were confirmed by computed tomography and/or magnetic resonance imaging. Transient ischemic attacks were diagnosed if the symptoms were self-limited in less than 24 hours, without residual neurological damage. Peripheral arterial disease was considered to be present if it was confirmed by Doppler and arteriography [5], [25].

To determine the potential association between the *IFNG* rs2430561 gene variant and the presence of subclinical atherosclerosis, between March 2007 and December 2010 a random subgroup of patients from the Lugo and Santander hospitals with no previous history of CV events was selected. In this regard, as previously reported, we assessed carotid ultrasonography studies in the common carotid artery to determine the carotid artery intima-media wall thickness (IMT) and presence of carotid plaques [26] in 286 patients. Also, an evaluation of endothelial function was assessed by a brachial artery reactivity study in 148 patients. Flow-mediated endothelium-dependent vasodilatation-FMD (post-ischemia) and endothelium-independent-NTG (post-nitroglycerin) vasodilatation were measured by brachial ultrasonography as previously described [26], [27].

**Table 6:** IFN-γ serum levels (pg/ml) in a subsample of patients (n = 153) with RA stratified according to the presence or absence of CV events.

	Serum IFN-γ levels, mean (SD)	*p*	*p* *
Patients without CV events (n = 81)	32.86 (81.93)		
Patients with CV events (n = 72)	57.54 (97.18)	0.09	0.04

* Analyses adjusted for gender, age at RA diagnosis, follow-up time from the disease diagnosis, presence or absence of shared epitope, and classic cardiovascular risk factors.

### *IFNG* Genotyping

Genomic DNA was isolated from whole blood using the QIAamp Blood DNA mini kit according to the manufacturer’s instructions (Qiagen lnc, Hilden, Germany). Allelic discrimination with TaqMan real-time PCR was used to genotype for polymorphism in the *IFNG* +874T/A gene with the following primers: forward 5′-TGCGAGTGTGTGTGTGTGT-3′ and reverse 5′-CAGACATTCACAATTGATTTTATTTTACAACACAAA-3′. Probe 1: 5′MGB- TGTGTGTGTGATTTGA 3′ –FAM and probe 2: 5′MGB- TGTGTGTGAGATTTGA 3′ -VIC (rs2430561). The PCR and end point analysis was performed in a volume of 4 µl containing 15 ng genomic DNA. The PCR was accomplished with the following amplification protocol: denaturation at 95°C for 10 minutes, followed by 45 cycles of denaturation at 95° for 15 seconds, and then annealing and extension at 60°C for 1 minute, in a 7900 HT Real-Time polymerase chain reaction (PCR) system, according to the conditions recommended by the manufacturer (Applied Biosystems, Foster City, CA, USA). Negative controls and duplicate samples were included to check the accuracy of genotyping.

**Table 7:** IFN-γ levels (pg/ml) in patients without CV events (left) or with CV events (right) according to genotypes and allele distribution of rs3430561 polymorphism.

*IFNG* +874T/A	without CV events, mean (SD)	*p*	*IFNG* +874T/A	With CV events, mean (SD)	*p*
TT (17)	11.47 (19.84)		TT (18)	99.90 (133.79)	
TA (42)	42.17 (103.65)		TA (36)	38.39 (79.17)	
AA (21)	33.13 (62.35)	0.44	AA (18)	53.49 (77.07)	0.09
TA+AA (63)	39.16 (91.53)	0.22	TA+AA (54)	43.42 (78.08)	0.03
T (76)	28.44 (79.23)		T (72)	69.15 (112.35)	
A (84)	37.65 (84.86)	0.48	A (72)	45.94 (77.41)	0.15

### Serum IFN-γ Levels Determination

Cytokine serum level was determined by Human IFN-γ Quantikine ELISA Kit (R & D Systems Europe, Ltd. Abingdon, UK) in 153 patients from Hospital Universitario Marqués de Valdecilla (Santander), following manufactureŕ instructions.

### Statistical Analysis

All genotype data were checked for deviation from Hardy-Weinberg equilibrium (HWE) using http://ihg.gsf.de/cgi-bin/hw/hwa1.pl. Comparison of proportions was carried out using χ^2^ test or Fisher test, when required. Strength of associations between CV events and genotypes or alleles of *IFNG* +874T/A gene polymorphism were estimated using odds ratios (OR) and 95% confidence intervals (CI), via logistic regression; estimates were further adjusted for sex, age at RA diagnosis, follow-up time, presence of anti-Cyclic Citrullinated peptide (anti-CCP) antibodies, and traditional CV risk factors (hypertension, diabetes mellitus, dyslipidemia, obesity and smoking habit) as potential confounders.

The association between genotypes of the *IFNG* functional variant and subclinical atherosclerosis markers or IFN-γ serum levels were tested using unpaired t test to compare between 2 groups, and one-way analysis of variance (ANOVA) to compare among more than two groups. Moreover, we also tested the association between these parameters and alleles using analysis of covariance (ANCOVA) adjusting for sex, age and duration of the disease at the time of the ultrasonographic study and traditional CV risk factors.

Statistical significance was defined as *p*≤0.05. All analyses were performed with STATA statistical software 9.1 (Stata Corp., College Station, TX, USA). Statistical power for the study was calculated using “CaTS - Power Calculator for Two Stage Association Studies” (http://www.sph.umich.edu/csg/abecasis/CaTS/) [28].

## Results

This multicentric study included 1635 RA patients. Information on the main demographical data, clinical characteristics of the RA patients enrolled in the current study, CV risk factors and CV events of patients is shown in **Table 1**. Among them, 332 (20.31%) experienced CV events: ischemic heart disease, heart failure, cerebrovascular accident or peripheral arteriopathy. With this sample size, the study had 73% statistical power to detect an OR equal to or higher than 1.25 in alleles present in 49% of the patients at the stated significance level (α = 0.05), type II error rate of 0.20 and a prevalence of the disease in Spanish population 0.005 [29]. The study reached a genotyping success >95.9%.

### Influence of the *IFNG* rs2430561 Functional Polymorphism in the Risk of CV Disease in RA Patients

Initially, we analyzed the genotype and allele distribution of the *IFNG* rs2430561 (+874T/A) polymorphism regarding the presence or absence of CV disease in RA patients (**Table 2**). Genotype frequencies were conformed to Hardy-Weinberg equilibrium (p>0.01). No differences in the frequency of A allele was found in patients who suffered or did not CV events (49.38% *vs.* 49.03%); neither statistically significant differences in the genotype and allele frequencies were found (*p* = 0.89, *p* = 0.87, respectively) when patients who had experienced CV disease were compared with those who had not suffered any kind of CV events.

### Logistic Regression Model to Explain the Presence of CV Disease in RA Patients According to *IFNG* rs2430561 Allele Distribution

In a further step, we constructed a logistic regression model analysis to explain the presence of CV disease according to the *IFNG* rs2430561 allele distribution, which was adjusted for classic CV risk factors, sex, age at the time of RA diagnosis, follow-up time from the disease diagnosis and anti-CCP status. No statistically significant differences on the risk of suffering CV events in this series of patients with RA were detected (*p* = 0.56, OR 1.08 [95% CI 0.84–1.39]) (**Table 3**). In addition, we assessed the influence of the variants in the occurrence of cardiac ischemic events, heart failure or cerebrovascular accidents. Still, no significant association was found (adjusted *p* = 0.74, OR 1.06 [95% CI 0.74–1.53]; *p* = 0.55, OR 0.88 [95% CI 0.59–1.32]; *p* = 0.56, OR 1.14 [95% CI 0.73–1.80], respectively). Besides, no significant associations were detected when we analyzed our cohort of patients divided by sex, anti-CCP status, or presence of hypertension, diabetes mellitus or dyslipidemia (data not shown).

**Table 8:** Comparison of IFN-γ levels in patients with or without CV events according to *IFNG* rs2430561 alleles in an adjusted ANCOVA model.

	With CV events	without CV events
rs2430561, *p* A vs. T*	0.052	0.58

* Analyses adjusted for gender, age at rheumatoid arthritis diagnosis, follow-up time from disease diagnosis, and classic cardiovascular risk factors: hypertension, diabetes mellitus, dyslipidemia, obesity and smoking habit.

### *IFNG* rs2430561 Functional Gene Polymorphism and Subclinical Atherosclerosis

Since the development and progression of atherosclerosis can be monitored using non invasive surrogate markers such as increased carotid artery intima-media thickness and presence of carotid plaques, we performed carotid ultrasonography to determine these parameters.

No statistically significant differences were observed among patients carrying the different genotypes or alleles of the *IFNG* rs2430561 (**Table 4**).

This study had >99% statistical power to detect a difference in carotid IMT of 0.1 mm or higher between T and A, or between TT and TA. Statistical power was >93% to detect a variation of 0.1 or higher in carotid IMT between TT and AA.

In the ANCOVA model adjusted for sex, age at the time of the ultrasonographic assessment, follow-up time, absence or presence of shared epitope and classic CV risk factors, no significant differences were found according to *IFNG* rs2430561 functional gene SNP (*p* = 0.63).

In keeping with results from carotid ultrasonography, no statistically significant differences were found in genotypes or alleles of patients who have carotid plaques *vs.* who did not have them in logistic regression model crude or adjusted by sex, age at rheumatoid arthritis diagnosis, follow-up time from the disease diagnosis, anti-CCP status and traditional CV risk factors (**Table 5**).

In our study, decreased FMD% were observed in RA patients carrying *IFNG* rs2430561 variant allele A (4.80 *vs.* 5.89), although the difference did not achieve statistical significance (*p* = 0.20), neither in the adjusted ANCOVA model (*p* = 0.11).

### *IFNG* rs2430561 Functional Variant and IFN-γ Serum Levels in RA Patients

The potential association between IFN-γ serum levels and CV events was assessed in 153 patients stratified according to the presence of CV events or not. As shown in **Table 6**, RA patients with CV events had higher levels of IFN-γ serum levels than those who did not experience CV events (adjusted *p* value = 0.039). However, no statistically differences were observed when patients without or with CV events were stratified according to *IFNG* rs2430561 genotypes (**Table 7**). Nevertheless, an ANCOVA model adjusted for sex, age at RA diagnosis, follow-up time from disease diagnosis, and classic CV risk factors, disclosed a marginally significant *p* value (0.052) when we compared the IFN-γ levels of patients carrying A allele with CV disease against patients who did not develop CV disease at the time of the study (**Table 8**).

## Discussion

To the best of our knowledge, this is the first study aimed to investigate the role of *IFNG* gene in the development of CV events in a population of RA patients. Our results do not support role of the *IFNG* rs2430561 (+874T/A) functional gene polymorphism in the increased risk of CV events in patients with RA.

The atherosclerotic lesion contains cytokines that promote a Th1 response (rather than a Th2 response). Activated T cells therefore differentiate into Th1 effector cells and begin producing the macrophage-activating cytokine IFN-γ. This cytokine improves the efficiency of antigen presentation and augments synthesis of the proinflammatory cytokines TNF-α and IL-1. Acting synergistically, these cytokines promote the production of many inflammatory and cytotoxic molecules in macrophages and vascular cells. All these actions tend to promote atherosclerosis [21]. With respect to this, it is known that patients with RA often have a “silent” history of coronary artery disease, with augmented frequency of myocardial infarction and sudden death [30].

Previous studies have described expression of IFN-γ by macrophages and endothelial cells within human atherosclerotic lesions [20]. Endothelial dysfunction in RA patients may persist in some patients with RA even when successful response to therapy is achieved [31]. In accordance with that, Kerekes *et al.* reported that serum levels of IFN-γ were significantly higher in patients with RA and low FMD values than in those with high FMD% values [32]. In our study decreased FMD were observed in RA patients carrying *IFNG* rs2430561 variant allele A, although the difference did not achieve statistical significance.

On the other hand, anti-TNF therapy is able to decrease the proportion of peripheral Th1, Th17 and IFN-γ-producing CD8+ T cells in RA patients [33], with simultaneous increase in the percentages of Tregs and, remarkably, in IFN-γ-producing NK cells.

Amezcúa-Guerra *et al.* described a subgroup of patients with aggressive RA that had high serum concentrations of IFN-γ and persistent endothelial dysfunction despite successful anti-arthritic therapy [31]. We also observed increased IFN-γ levels in patients with CV events in our study. However, no statistically significant association between serum levels of IFN-γ and *IFNG* rs2430561 genotypes was found in our series, although the adjusted ANCOVA model disclosed a marginally significant *p* value when we compared de IFN-γ levels of patients carrying A allele with CV disease against patients who did not develop CV disease at the time of the study. The AA homozygote for *IFNG* +874T/A polymorphism was associated with adverse outcome, worse prognosis as well as with measures of disease severity in idiopathic dilated cardiomyopathy in a recent study [34].

Intriguingly, patients with RA exhibit expansion of the Th1-CD4+CD28_null_ cell population in the peripheral blood, which correlates with higher frequency of extraarticular manifestations, increased carotid IMT, and decreased FMD% values, supporting the concept that CD4+CD28_null_ cells may sustain synovial inflammation and promote atherosclerosis in these patients [35], as they have been implicated in atherosclerotic plaque disruption [36] and in all phases of atherosclerotic process [37]. Our results of endothelial dysfunction although not reaching statistical significance, are in agreement with those studies and observations. However, we have not been able to demonstrate association between *IFNG* +874T/A functional polymorphism and the risk of suffering CV events in Spanish RA patients.

Nevertheless, it is possible that besides genetic influence, additional factors may influence IFN-γ levels, as immune response in RA may be the result of a complex network of pro- and anti-inflammatory mediators. Taken together all these observations, it is possible that *IFNG* along with other genes implicated in the inflammatory response may contribute to the development of the high-grade systemic inflammation status seen in RA patients that could explain the increased CV morbidity and mortality reported in these patients. In addition, it is likely that the relatively low statistical power of the study to detect small effects may explain, at least in part, our negative results.

In summary, our results do not show a direct influence of the *IFNG* rs2430561 (+874T/A) functional gene polymorphism in the increased risk of CV events observed in patients with RA. Further studies need to be carried out to enlighten the role of this gene in the predisposition to CV disease in RA patients.
